# Age- and Sex-Based Developmental Biomarkers in Eye Movements

**DOI:** 10.3390/brainsci14121288

**Published:** 2024-12-21

**Authors:** Frederick Robert Carrick, Melissa Hunfalvay, Takumi Bolte, Sergio F. Azzolino, Mahera Abdulrahman, Ahmed Hankir, Matthew M. Antonucci, Nouf Al-Rumaihi

**Affiliations:** 1 College of Medicine, University of Central Florida, Orlando, FL 32827, USA; 2 Department of Neurology, The Carrick Institute, Cape Canaveral, FL 32920, USA; melissa@righteye.com (M.H.); sergio@azzolino.com (S.F.A.); akzhankir@gmail.com (A.H.); mantonucci@carrickinstitute.com (M.M.A.);; 3 Centre for Mental Health Research in Association with the University of Cambridge, Cambridge CB2 1TN, UK; 4 Burnett School of Biomedical Science, University of Central Florida, Orlando, FL 32827, USA; 5 RightEye LLC, 6107A, Suite 400, Rockledge Drive, Bethesda, MD 20814, USA; takumi@righteye.com; 6 Biological Engineering, Massachusetts Institute of Technology, Cambridge, MA 02139, USA; 7 Department of Informatics and Smart Heath, Dubai Health Authority, Dubai 431111, United Arab Emirates; dr.m.abdulrahman@gmail.com; 8 Department of Public Health, Mohammed Bin Rashid School of Medicine, Dubai 88905, United Arab Emirates; 9 School of Medicine, Cardiff University, Cardiff CF14 4YS, UK; 10 Schulich School of Medicine and Dentistry, University of Western Ontario, London, ON N6A 5C1, Canada; 11 Saudi Commission for Health Specialties, Riyadh 11614, Saudi Arabia

**Keywords:** eye tracking, gender, age, visual saccades, visual pursuits, neurology, neuro-otology, functional neurology, normative data

## Abstract

Background: Eye movement research serves as a critical tool for assessing brain function, diagnosing neurological and psychiatric disorders, and understanding cognition and behavior. Sex differences have largely been under reported or ignored in neurological research. However, eye movement features provide biomarkers that are useful for disease classification with superior accuracy and robustness compared to previous classifiers for neurological diseases. Neurological diseases have a sex specificity, yet eye movement analysis has not been specific to our understanding of sex differences. Methods: The study involved subjects recruited from 804 sites equipped with RightEye Vision Systems, primarily located in optometry practices across the United States. Subjects completed six eye movement assessments: circular smooth pursuit (CSP), horizontal smooth pursuit (HSP), vertical smooth pursuit (VSP), horizontal saccades (HS), vertical saccades (VS), and fixation stability (FS). Eye movements were analyzed and classified in accordance with age and sex by multiple *t*-tests and linear regression models. Results: This study represented a large sample size of 23,557 subjects, with 11,871 males and 11,686 females representing ages from birth through 80 years of age. We observed statistically significant differences for all eye movement functions between males and females. Conclusions: We demonstrate that eye movements are sex-specific and offer normative data to compare sex-specific eye movement function by age. Novel baseline metrics can be compared to individual performance, regardless of sex. This study represents significant progress in linking eye movements with brain function and clinical syndromes, allowing researchers and clinicians to stratify individuals by age and sex.

## 1. Introduction

Neurological diagnosis relies on subjective symptom evaluation, which can be different depending on the clinician’s experience. There is a need to establish objective biological measures that can complement clinician experience and support diagnostic and therapeutic decision-making. Obtaining normative data specific to females in medical science is crucial for ensuring accurate diagnosis, effective treatment, and equitable healthcare outcomes. Historically, the majority of medical research and clinical trials have been conducted predominantly on male subjects, leading to diagnostic criteria, treatment guidelines, and medical devices that may not account for sex-specific physiological and hormonal differences. For example, women often present with different symptoms for many conditions, which can lead to misdiagnosis or delayed care when using male-centric standards. Additionally, pharmacokinetics and drug metabolism can vary between sexes, influencing medication efficacy and risk of side effects. Conditions such as autoimmune diseases, osteoporosis, and gynecological health issues also require female-specific normative data to better understand their prevalence, progression, and optimal management. By gathering and analyzing normative data for females, the clinical community can create more personalized and inclusive care strategies that address the unique health needs of women. Among potential biomarkers for a variety of neurological conditions and diseases, eye movement abnormalities have emerged as promising potential biomarkers for a variety of neurological conditions and diseases. The structure and function of the brain are significantly influenced by both age and sex. Despite this, neurological research often treats age and sex as confounding factors, frequently combining male and female subjects in both human and animal studies. Eye movements are sensorimotor functions regulated by neural mechanisms that may exhibit variations based on age and sex. A clear understanding of these influences is crucial to differentiate disease-specific abnormalities from natural variations linked to aging or sex differences. Aging affects certain eye movement characteristics, such as reducing saccade velocity, decreasing visual tracking performance, and impairing antisaccade performance in older individuals. However, we do not have evidence that is sex-specific, and we are not aware of studies that report the influence of age and sex on eye movements in large adult populations. The absence of sex-delineated research biases clinicians and limits the reproducibility and generalizability of findings to the general population. To address these gaps, we wanted to explore the effects of age and sex on eye movement metrics in a large cohort of healthy adults. Our aim was to provide valuable insights into age- and sex-dependent variations in eye movement characteristics that might establish normative data for future neurological research.

### Background

Our group identified when and how eye movements change across the human lifespan to benchmark developmental biomarkers in a large sample size of 45,696 subjects using a retrospective analysis with machine learning [1]. Like most studies, we did not control for sex, and combined males and females. We identified 23,557 subjects (11,871 males and 11,686 females) classified by sex. We did not analyze eye movements specific to sex in that large study or in other recent investigations [2,3,4,5,6,7,8,9]. However, a small study on sex-related eye movement differences found that females exhibited higher global oculomotor activity [10] and we used the 23,557 subjects identified by sex in the large 45,696 subject study to explore differences between male and female oculomotor activity across the lifespan. There are sex differences in visuomotor tracking, revealing a male advantage in eye hand-tracking accuracy attributed to faster decision-making processes linking visual input to hand movements, despite similar gaze strategies and hand kinematics between sexes [11]. Men and women demonstrate distinct navigation performances and gaze patterns, with men favoring Euclidean strategies and while women relied on landmarks influenced by estradiol levels, showing longer gaze distances than men [12]. While men and women allocate visual attention similarly during mental rotation tasks, men demonstrate greater discrimination and a longer processing of correct alternatives, with androgen levels influencing task-relevant fixation patterns in men and visual persistence in women, highlighting hormonal contributions to sex differences in cognitive processing [13]. Accurate and low variability sex prediction can be made via eye movements, reconfirming that females exhibit a stronger bias toward the left eyes of facial stimuli [14]. Sex differences in exploratory eye movements are characterized by longer gazing times and shorter scanning lengths in adult women compared to men and are evident only during adulthood, suggesting a regulatory role of gonadal hormones in visual information processing [15]. Older adults show higher total eye-scanning lengths (TESL) and gaze points (TNGP) than younger groups while sex differences appear to be limited to young adults suggesting hormonal influences [16]. However, one study found that multiple step saccades (MSS) increase with age, differ between vertical and horizontal saccades, and are distinct from corrective saccades (CS), with no sex differences observed, highlighting age and saccadic direction as key factors influencing saccadic behavior and their potential as indicators of brain function affected by aging [17]. We know that eye tracking can quantify sex differences in landmark utilization, demonstrating that women rely more on landmarks than men who exhibit reduced landmark-oriented gaze over time [18]. We are aware of one study that found significant sex differences in exploratory eye movements, with women showing longer mean gazing times and fewer gazing points than men, while men exhibited longer total eye-scanning lengths, suggesting differences in visual information processing and confirming the reproducibility of these parameters over time [19]. It is accepted that age significantly impacts eye movement measures, particularly those related to fixation and the motor control of smooth pursuit and saccades with high reproducibility, whereas sex effects are less consistent, emphasizing the importance of accounting for age in evaluating eye movement abnormalities [20]. We were concerned that the lack of consistency in sex differences in eye movements were probably due to small sample sizes and the absence of studies that addressed sex-specific normative data across the lifespan. For instance, eye-tracking strategies after sleep deprivation were not able to assess sex due to unequal group distribution [21]. Females have superior face recognition skills driven by variations in saccade paths rather than fixation frequency within regions of interest (ROIs), with females showing increased transitions to the eyes and utilizing saccade path dynamics that can differentiate females from males [22]. Our review of the literature and our clinical experience suggested that there must be differences in eye movements between males and females. We needed a larger sample size than all previous studies to compare the eye movements of females to males.

## 2. Materials and Methods

### 2.1. Participants

The study involved participants recruited from 804 sites equipped with RightEye Vision Systems, primarily located in optometry practices across the United States. Participants were individuals attending these practices for clinical visits or annual examinations. Eligibility criteria required participants to have completed six eye movement assessments: circular smooth pursuit (CSP), horizontal smooth pursuit (HSP), vertical smooth pursuit (VSP), horizontal saccades (HS), vertical saccades (VS), and fixation stability (FS). Exclusion criteria included the presence of eyelash impediments, the recent consumption of drugs or alcohol within 24 h prior to testing, a positive diagnosis of strabismus, or failure to meet all nine calibration points. The final sample comprised 23,557 participants aged 6 to 80 years. Of this sample, 11,871 were male (mean age 28.7 ± 16.7) and 11,686 were female (mean age 31.3 ± 18). Data on race and ethnicity were reported by 42.38%. Among those, 69.53% identified as White, 5.48% identified as Asian, 5.07% identified as Latin American, and 4.11% identified as Black.

### 2.2. Ethical Guidelines

This study was conducted in compliance with the ethical principles outlined in the Declaration of Helsinki and was approved by the Institutional Review Board of East Carolina University (IRB UMCIRB 13-002660). All data used in the analysis were fully anonymized to protect participant confidentiality.

### 2.3. Testers

Testing was carried out by optometrists certified by the American Board of Optometry. These clinicians completed specialized training on the operation and application of the RightEye Vision System, subsequently becoming certified RightEye providers.

### 2.4. Apparatus

The assessments were conducted using the RightEye Vision System, which presented stimuli on a Tobii I15 Vision monitor integrated with a 90 Hz Tobii remote eye tracker. Input devices included a Logitech wireless keyboard and mouse (model Y-R0017, Logitech, San Jose, CA 95134, USA). The Tobii eye tracker offered an angular accuracy of 0.4°, operating within a headbox measuring 32 cm × 21 cm and positioned at an optimal viewing distance of 55 to 60 cm from the display screen.

### 2.5. Testing Procedure

Optometrists completed the RightEye Basic Training Course, which covered standardized procedures for test setup and data collection. The testing environment was a quiet and private space, with participants seated in a stationary, height-fixed chair without wheels to maintain consistent positioning. Participants were placed at a desk with their heads left unconstrained throughout the procedure to allow natural movement. To maintain standardization, participants were seated at a precise distance of 56 cm from the eye-tracking monitor. This positioning, which fell within the optimal range of the Tobii eye tracker’s headbox, was verified in real time using the RightEye headbox guidance system. Participants underwent a standard 9-point calibration procedure to ensure accurate alignment of the eye-tracking system. Only individuals who successfully completed all nine calibration points were subsequently administered the oculomotor tasks.

### 2.6. Oculomotor Tasks

Calibration Task: Participants completed a 9-point calibration task designed to align the eye-tracking system accurately. Each calibration point was 1° in size and displayed individually for 2 s at random screen locations. The task, lasting 18 s, ensured the participant’s gaze covered nine distinct screen regions. This calibration allowed the eye tracker to convert pixel coordinates of the pupil center and corneal reflection into gaze locations on the screen. The resulting data included pupil measurements, such as pupil size, and were used to track gaze during subsequent oculomotor assessments.

Pursuit Tasks: Pursuit tasks included circular smooth pursuit (CSP), horizontal smooth pursuit (HSP), and vertical smooth pursuit (VSP). Participants were instructed to “follow the dot on the screen as accurately as possible with your eyes”. The dot, measuring 0.2° in diameter, moved at a velocity of 25.13° of visual angle per second against a black background with white dots. Each task lasted 20 s. For the CSP test, the dot moved in a circular trajectory with a 20° diameter. For the HSP test, the dot oscillated 15° left and right from a central point, covering a total horizontal range of 30°. In the VSP test, the dot moved 11° upward and 11° downward from the center, spanning a vertical range of 22°.

Saccade Tasks: The horizontal (HS) and vertical (VS) self-paced saccade tasks required participants to visually alternate between two target dots. Each test began with a 3-2-1 countdown displayed at the screen’s center. Participants were instructed to “target each dot” as quickly and accurately as possible, with the HS test involving horizontal movements between left and right dots and the VS test requiring vertical movements between upper and lower dots. Each task was conducted over a 10 s duration.

Fixation Task: The fixation stability (FS) test involved viewing three distinct targets, each presented for 7 s, with a 3 s interlude between presentations. Standardized verbal instructions were given before each target: “Move your eyes to the center of the target. Keep your eyes as still as possible until the target disappears”. Participants confirmed their readiness by responding “Yes” after being asked, “Are you looking at the center of the target?” The tester initiated the task by pressing the spacebar. The targets included a 1° cross, a 1° filled circle, and a 4-point diamond (3° point-to-point separation), with dimensions consistent with the Humphrey Field Analyzer (Carl Zeiss Meditec, Dublin, CA, USA) [23].

In summary, the tasks encompassed all primary types of eye movements utilized by clinicians, including fixations, saccades, pursuits, and vergence.

### 2.7. Statistical Analysis

Statistical Analysis was carried out in STATA 18.5, College Station, TX 77845, USA and included multiple *t*-tests and a multiple regression analysis of the data with statistical significance if an alpha of less that 0.05 was met with 95% confidence.

## 3. Results

Table 1 summarizes the eye movements by age and sex. The results of eye movements are shown as *n* (%), mean ± SD, or mean ± SD [min–max] with statistical significance. Table 2 delineates the technical definition and measurement of all variables listed in Table 1.

Multiple *t*-tests were made comparing the differences in eye movement performance between males and females by age (Table 3).

With 23,557 subjects, a scatterplot that plots every data point would become too crowded to interpret visually. We therefore grouped the x-axis variable into equal-sized bins and computed the mean of the x-axis and y-axis variables within each bin, then created a scatterplot of these data points. By including all the data in each scatterplot, we found that this method was better than taking random samples of the data and plotting them. The result is a non-parametric visualization of the conditional expectation function.

**Figure 1:**
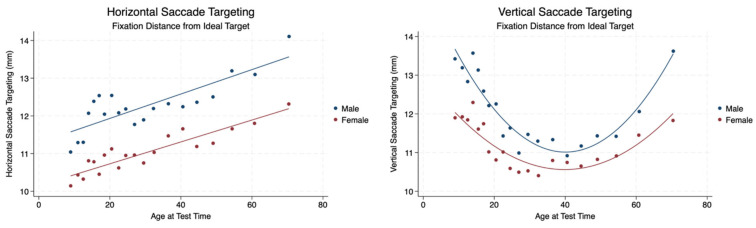
Fixation distance from ideal target during saccades by sex and age.

We wanted to quantify where the eye was in relation to the target during saccades in the horizontal and vertical planes. We analyzed the distance between each fixation and the location of the actual target. We found a statistically significant difference with a mild effect size between male and female horizontal and vertical saccadic targeting. Females performed better than males with a fixation target closer to the ideal target. The distance between the eye fixation points and the target increased when performing horizontal saccades over the lifespan, similarly for both males and females in a linear fashion. However, males and females demonstrated improved accuracy with a decrease in the distance of visual fixation from the target at approximately 40 years of age when performing vertical saccades. Female performance was superior to male performance in vertical saccade targeting. Both males and females older than 40 years of age demonstrated that the distance between eye fixation points and target location gradually increased. The increase and decrease in fixation distance from an ideal target in the vertical plane can be visualized as a U-shaped curve, quite different from the linear relationship of horizontal saccade targeting (Table 3, Figure 1).

**Figure 2:**
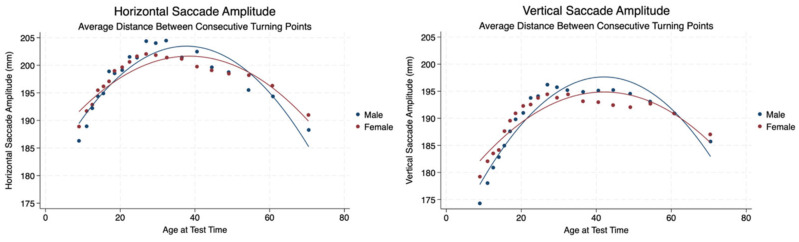
Amplitude of saccades in the horizontal and vertical planes by sex and age.

We calculated the average distance between consecutive turning points for horizontal and vertical saccadic eye movements to ascertain the amplitude of the eye movements. We found a progressively increasing amplitude to fixed turning points until approximately 38 years of age, and then the amplitude decreased throughout the remaining lifespan. There was no significant difference in the amplitudes registered between sexes, and the effect sizes were negligible (Table 3, Figure 2).

**Figure 3:**
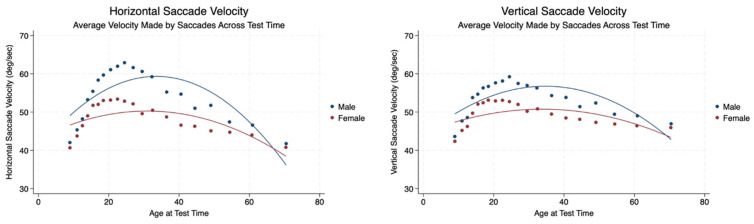
Velocity of saccades in the horizontal and vertical planes by sex and age.

We measured the average velocity made by the saccades across the test time in both the horizontal and vertical planes. There was a statistically significant difference in saccadic velocity between males and females with mild to moderate effect sizes. Males made higher velocity saccades than women in both planes. The velocity of saccades increased up to approximately 20 years of age for both sexes and then decreased gradually through the remainder of the lifespan (Table 3, Figure 3).

**Figure 4:**
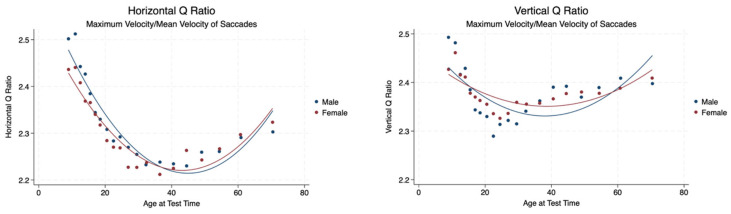
Horizontal and vertical Q ratios by sex and age.

The Q ratio is the ratio of the peak velocity to average velocity in the saccadic interval. There is a statistically significant difference between the Q ratio in the horizontal plane between males and females with a mild effect size. There was no significant difference between male and female Q ratios in the vertical plane with a mild effect size. There was a statistically significant difference between the horizontal and vertical Q ratios (*p* < 0.001) with a mild effect size (Cohen’s d = 0.19) (Table 3, Figure 4).

**Figure 5:**
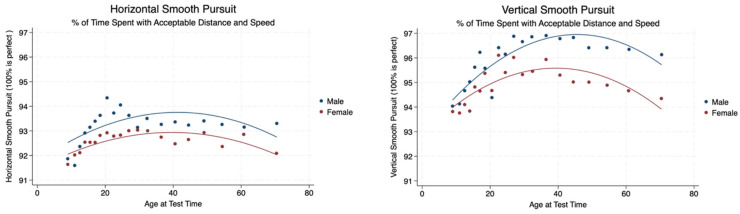
Smooth pursuit measured as an acceptable distance and speed in the horizontal and vertical planes by sex and age.

We measured the percentage of time spent in smooth pursuit with an acceptable distance and speed measured as a percentage (100% represented a perfect performance). There was a statistically significant difference between the performance of males and females with males performing better in both the horizontal and vertical planes with mild effect sizes. Males and females demonstrated a gradual increase in performance until the age of 30 years, followed by a steady state performance throughout the remainder of life in both planes. The performance of smooth pursuit in the vertical plane was superior to that in the horizontal plane with a statistically significant difference between smooth pursuit performance (*p* < 0.001) and a mild effect size (Cohen’s d = 0.19) (Table 3, Figure 5).

**Figure 6:**
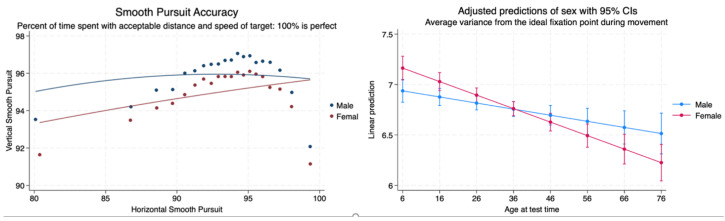
Smooth pursuit accuracy relationship between horizontal and vertical smooth pursuits and the average variance from the ideal fixation point during eye movements.

We compared the accuracy of horizontal compared to vertical smooth pursuits for males and females. There was a significant difference between the sexes, with vertical smooth pursuit accuracy shown to be better for males when comparing them to horizontal smooth pursuit accuracy. There was a gradual increase in vertical smooth pursuit accuracy for males and females as horizontal smooth pursuit accuracy increased to approximately 95%, and then there was a dramatic decrease in accuracy for the remainder of the lifespan. A linear regression of the average variance from the ideal fixation point during movement in both horizontal and vertical planes demonstrated that females performed worse than males until the age of 36 when they demonstrated less variance and better performance than males for the remainder of the lifespan (Table 3, Figure 6).

**Figure 7:**
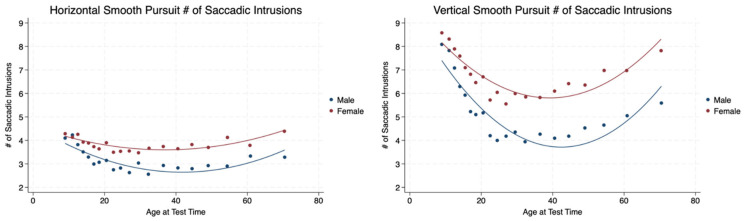
Saccadic intrusions made during pursuits in the horizontal and vertical planes by sex and age.

We counted the number of saccadic intrusions made during smooth pursuits across the test time in the horizontal and vertical planes. Females made more saccades during pursuits than males with statistical significance and a moderate effect size. The number of saccades made in the horizontal plane were stable for both sexes during the lifespan in the horizontal plane. The number of saccades made during pursuits was greater in the vertical plane compared to the horizontal plane with statistical significance (<0.001) and a moderately strong effect size (Cohen’s d = 0.66). There was a marked decrease in saccadic intrusions for both sexes until the age of 20 years with a rather stable number of saccades made during pursuits after the age of 20 for both males and females (Table 3, Figure 7).

**Figure 8:**
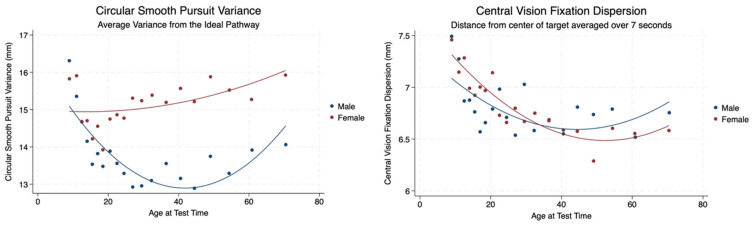
Circular smooth pursuit variance and central vision fixation dispersion by sex and age.

We analyzed the ability of subjects to follow a target that made a circular pathway and recorded the average variance from the ideal circular pathway. Females had a statistically significant greater variance from a circular pathway with a mild effect size compared to males. Females demonstrated a gradual increase in variance over the lifespan. Males, however, decreased their variance from a circular pathway to approximately age 40 and then had a gradual increase throughout the rest of the lifespan. We also observed the distance from the center of a target (central vision fixation dispersion (CVFD)) averaged over 7 s during smooth pursuits. Although there was no significant difference between males and females, there was an interesting pattern of CVFD observed. The females had a greater CVFD than males, which decreased until the age of 35 years when males increased their CVFD and continued to increase it gradually throughout the remainder of the lifespan. The females continued to decrease their CVFD after 35 years of age until age 60 when they stabilized their distance from the center of the smooth pursuit target (Table 3, Figure 8).

**Figure 9:**
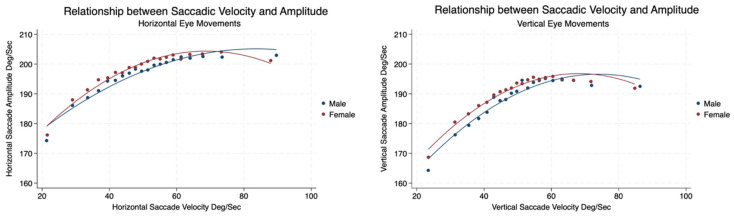
Relationship between saccadic velocity and amplitude associated with horizontal and vertical eye movements by sex and age.

We found that there was no significant difference in the relationship between the amplitude and velocity of saccades made by males and females performing horizontal and vertical planes. Horizonal amplitudes increased when horizontal saccadic velocities increased gradually to the age of 75 and then decreased moderately throughout the remainder of the lifespan, with females demonstrating a greater decrease than males. The same pattern was observed from the increase in vertical saccadic amplitude as the vertical horizontal saccadic velocity increased; however, both the velocity and amplitude of vertical saccades were less than those made in the horizontal plane (Table 3, Figure 9).

Multiple regression models of eye movements were made to predict the age of participants for all eye movements by sex and summarized in Table 4.

**Figure 10:**
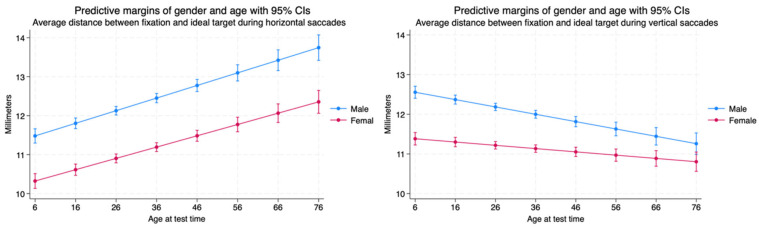
Average distance between fixation and ideal target during saccades.

We found that there was a statistically significant difference between the average distance between fixation and the ideal target during horizontal saccades between males and females. The females performed better than males, but both sexes demonstrated a decline in saccadic targeting, with an increase in the distance between fixation and ideal targets over the lifespan while performing horizontal saccades. When performing vertical saccades, females were also better than males. However, there was an increase in accuracy throughout the lifespan when performing vertical saccades in contrast to the decrease in accuracy observed in the horizontal plane. The average distance between fixation and the ideal target becomes lower much more dramatically for males than females, but female performance was better throughout the lifespan (Table 4, Figure 10).

**Figure 11:**
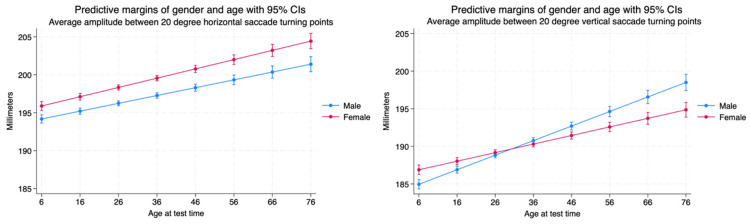
Average amplitude between 20 degree horizontal and vertical saccade turning points by age and sex.

There was a significant difference between the performance by males and females of the average amplitude between 20 degree horizontal saccade turning points. The amplitudes of female saccades were greater than those made by males. Both sexes demonstrated an increase in the amplitude of saccades made in the horizontal plane throughout the lifespan. Saccadic amplitude performance in the vertical plane is different between sexes than in the horizontal plane. The difference in performance between males and females is significant but the amplitude of the saccades is much less than that observed in the horizontal plane. Although both males and females demonstrate an increase in vertical saccadic amplitude performance throughout the lifespan, the female amplitudes are greater than males up the age of 20, and the male amplitudes become larger than those made by females. This relationship is maintained for the remainder of the lifespan (Table 4, Figure 11).

**Figure 12:**
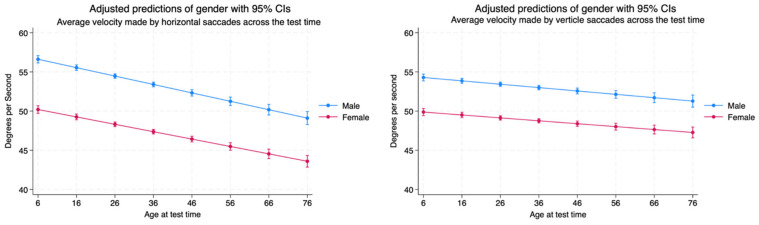
Average saccadic velocity made in horizontal and vertical planes by age and sex.

The average velocity of horizontal saccades is statistically higher for males than females. The saccadic velocity in the horizontal plane decreases over the lifespan. The velocity of the saccades made in the vertical plane is significantly higher for males as observed in the horizontal plane. The saccades do decrease in velocity over the lifespan, but they do not degrade at the same rate as those made in the horizontal plane and maintain their early age-related amplitudes well as the subjects age (Table 4, Figure 12).

**Figure 13:**
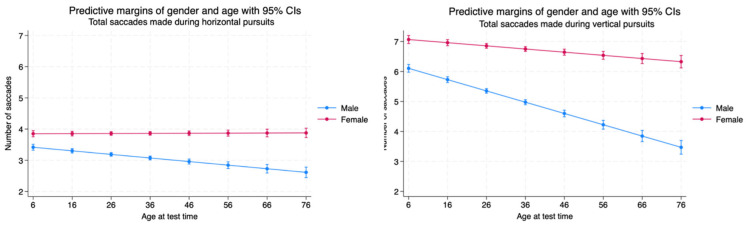
Total saccades made during horizontal and vertical pursuits by age and sex.

Males made significantly less saccades during horizontal pursuits than females and they decreased the number of saccadic intrusions gradually during the lifespan. Females maintained a higher number of saccadic intrusions than males and showed no increase or decrease in the number of saccades generated over the lifespan. The number of saccadic intrusions made in the vertical plane of pursuits was significantly greater than those made during the horizontal plane. Females made significantly more saccades during vertical pursuits than males. Although the females made less saccades during vertical pursuits over their lifespan, the number of intrusions was maintained well, with a decrease in number of only 0.5 saccades in the mid 70s from that observed in early age. Males had an improvement of vertical pursuit integrity with approximately three decreased saccadic intrusions by the mid 70s (Table 4, Figure 13).

**Figure 14:**
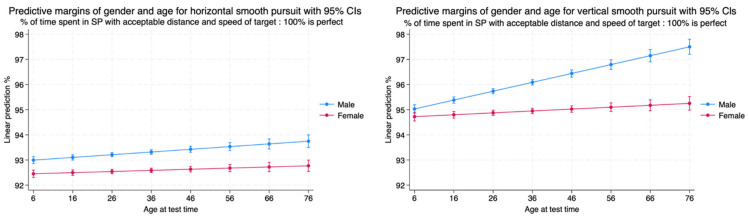
Percentage of time spent in smooth pursuit in the horizontal and vertical planes with an acceptable distance and speed of the target by age and sex.

Males made their horizontal smooth pursuits with an increased probability of having an acceptable distance from a target. This was significantly different and better than the smooth pursuit demonstration of accuracy in the horizontal plane by females. Male accuracy did improve over the lifespan but by only 0.5% in contrast to female performance, which did not really change over the lifespan. The accuracy of pursuits made in the vertical pane was significantly better than their performance in the horizontal plane as well as better than female performance. The male vertical pursuits became impressively better over the lifespan, while the female pursuits were rather stable over the lifespan (Table 4, Figure 14).

## 4. Discussion

Eye movement research serves as a critical tool for assessing brain function, diagnosing neurological and psychiatric disorders, and understanding cognition and behavior. It also provides objective biomarkers for disease progression, phenotypic classification, and therapy monitoring, emphasizing the need for standardized ocular motor testing [24]. Sex differences have largely been under reported or ignored in neurological research. Yet, eye movement features provide biomarkers that are useful for disease classification with superior accuracy and robustness compared to previous classifiers for neurological diseases [25]. Neurological diseases have a sex specificity, yet eye movement analysis has not been specific to our understanding of sex differences. Our study of eye movements classified by sex and age should assist in the classification of neurological diseases in a more realistic fashion.

We can now apply sex-specific data to a physiologically motivated framework for the model-based separation, detection, and classification of eye movements. We enable the accurate analysis of kinematic and neural signals across various eye movement types, thus offering potential for identifying novel digital biomarkers for both males and females [26]. Sex-specific eye movement data will complement eye-tracking and machine-learning classifiers in detecting Parkinson’s disease (PD) with high sensitivity and specificity for sex differences and understanding of the cognitive dysfunction stages that have not been made available for females [27]. Smooth pursuit eye movement abnormalities, characterized by reduced gain and saccadic pursuit are prevalent in PD, correlate with disease severity, and may serve as biomarkers for diagnosis and progression [28]. However, our findings demonstrate that smooth pursuit eye movement disorders are different for males and females and biomarkers must be adjusted to sex.

Other common neurological syndromes such as traumatic brain injury (TBI) significantly impair oculomotor functions that serve as sensitive biomarkers for neural and cognitive deficits in TBI [29]. The addition of sex-specific eye movement classifiers found in our study should contribute to a more realistic view of brain function and deficits after TBI. Athletes with sport-related concussion (SRC) exhibit larger, faster saccades but slower smooth pursuit eye movements during a sport-like task [30]. The differences in the sex performance of saccades and pursuits matched by age can serve as a reference of normative data from which SRC can easily be quantified for males and females. Modern eye-tracking technology can also identify visual processing impairments and saccadic dysfunctions as non-invasive biomarkers for schizophrenia, emphasizing its role in early intervention and refining diagnostic approaches [31]. Eye-tracking paradigms also address limitations in traditional cognitive assessments, offering non-invasive and cost-effective biomarkers for the early detection of mild cognitive impairment (MCI) and Alzheimer’s disease (AD) [32]. The necessary inclusion of sex-specific eye movement data should increase the diagnostic and therapeutic applications to schizophrenia.

There is incredible potential to develop reliable, low-cost, and non-invasive methods for early detection of Alzheimer’s and Parkinson’s diseases, with sex-specific data that will greatly contribute to the validation of findings [33]. Ocular biomarkers for early AD and MCI have shown moderate diagnostic accuracy due to limitations in study design and reporting that underscore the need for longitudinal studies to improve predictive value [34]. The inclusion of longitudinal sex- and age-specific normative data, as in this study, should contribute to the improvement of predictive value. Combining eye movement metrics, demographics, and cognitive test scores provides a non-invasive, cost-effective method for predicting mild cognitive impairment (MCI), achieving strong classification performance and highlighting associations with attentional and executive function deficits [35]. We expect that the inclusion of sex-specific eye movement metrics will increase the ability to predict MCI between males and females and decrease the historical sex bias that has confounded diagnostic and therapeutic approaches to attentional and executive function deficits.

Eye-tracking in adults can identify potential biomarkers for depressive disorders, using robust quality and evidence assessment methods [36]. Our findings may promote the ability to identify sex-specific biomarkers that were not available and are associated with depressive disorders. For example, there are sex-related risk pathways for post-traumatic stress disorder (PTSD), showing that acute stress disorder, neuroticism, lifetime sexual assault, anxiety sensitivity, and pre-trauma anxiety fully mediate the relationship between sex and PTSD severity, highlighting the importance of sex-sensitive approaches in mental health research and interventions [37]. Eye-tracking can assist these approaches in a non-invasive sex-specific fashion. Other common mental health concerns such as postpartum depression (PPD), a prevalent and serious public health issue that often goes undiagnosed and untreated in primary care, leaves many affected mothers and their families without necessary support [38]. Eye movement metrics are cost-effective non-invasive biomarkers for diagnosing depression [39]. Our findings should complement the diagnosis of depression that are sex-specific such as PPD.

There is a link between cerebellar vermis structure and saccadic eye-movement plasticity in autism spectrum disorder (ASD), suggesting that vermal hypoplasia may define a subphenotype characterized by severe visuo-sensorimotor and social deficits, enabling more targeted diagnosis and interventions [40]. Autism is significantly more common in boys than in girls, suggesting that the normative sex differences in saccades that are identified in this study may provide extremely useful diagnostic biomarker criterion in ASD. Children with ASD exhibit significantly reduced gaze fixation to the eye region of faces, suggesting it as a potential biomarker, while findings on mouth fixation remain inconclusive due to study heterogeneity [41]. In addition, a recent systematic review and meta-analysis found that increased gaze toward faces, head, and eye regions is associated with improved social functioning and reduced autism symptom severity in ASD, highlighting gaze variables as potential biomarkers [42]. The differences in eye movement performance between sexes can now be used to define a probability of ASD when the spread between performance increases, directing attention to both lower and higher sex-based performances.

Smooth pursuit eye movement impairments are observed in up to 80% of schizophrenia patients and may serve as potential biomarkers linked to neuroanatomical and neuropsychological features of the disease [43,44]. However, sex influences the epidemiology, presentation, risk factors, and management of schizophrenia, with biological and reproductive differences in women affecting disease onset and progression, underscoring the need to address sex-related factors in advancing precision psychiatry [45]. Having a normative database of eye movements specific to age and sex is surely associated with a greater understanding of schizophrenia and other mental health disorders that have a sex bias.

We controlled for recent drug/alcohol consumption and strabismus, but these are not the only sources of variation in eye movements. We have addressed the myriad of possible conditions that might affect eye movements in our Section 4 but did not control for them in our analysis as we had no indication that any of these were involved in our sample. We utilized machine learning approaches to assist in identifying latent relationships among variables specific to eye movements across the human lifespan in a large sample size of 45,696 subjects from which this sample was drawn [1]. These methods use patterns in the data to suggest confounding variables even without prior assumptions. We found no indication of confounding factors.

## 5. Conclusions

Many early-stage diseases are difficult to identify, but non-invasive biomarkers such as eye movements offer a promising solution. Our study demonstrates that eye movements are sex-specific and offer normative data to compare function. Eye movement analysis provides baseline metrics that can be compared to individual performance, regardless of sex, enabling the early detection of deviations associated with many diseases and neurodegenerative disorders. An early diagnosis often improves prognosis, treatment outcomes, and survival rates, while the non-invasive nature of eye tracking enhances patient comfort and compliance. Eye tracking is cost-effective and requires fewer resources than traditional diagnostic methods, which makes it accessible even in resource-limited settings.

This study represents significant progress in linking eye movements with brain function and clinical syndromes, allowing researchers and clinicians to stratify individuals by age and sex. This research identifies age–sex-specific biomarkers, showing that eye movement efficiency improves until early adulthood and declines with aging, with distinct patterns emerging in younger and older populations. These insights enhance the understanding of cognitive and neural development across the lifespan with elucidation of sex differences. Eye movement biomarkers also hold potential for the early detection and monitoring of neurological and cognitive disorders that might be sex biased, offering clinicians a tool to track disease progression and treatment efficacy. For example, abnormalities in eye movements may indicate early stages of Parkinson’s disease or dementia.

The ability to quantify age and sex performance of eye movements can improve the design of personalized assistive technologies and clinical trials by accounting for variations in eye movement behavior, thereby increasing the reliability of research findings and clinical applications. Individuals can now be categorized into age and sex groups with remarkable accuracy based on eye movement data, supporting the development of precision medicine. The biomarkers identified in this study enable real-time monitoring, patient-specific interventions, and the more effective management of age–sex-related diseases. Integrating eye movement biomarkers into clinical practice can transform healthcare by improving early diagnosis, reducing costs, and enhancing patient outcomes across the lifespan. Continued innovation in this field is critical to advancing medical science and ensuring a better quality of life for patients worldwide.

## Figures and Tables

**Table 1:** Eye movements by age and sex.

	Male	Female
	(*n* = 11,871)	(*n* = 11,686)
Age at Test Time	28.7 ± 16.7	31.3 ± 18.0
Horizontal Saccade Targeting	12.2 ± 6.5	11.1 ± 5.6
Horizontal Saccade Amplitude	197.4 ± 19.2	197.3 ± 18.0
Horizontal Saccade Velocity Averaged	54.2 ± 17.1	47.8 ± 13.6
Vertical Saccade Targeting	12.1 ± 5.3	11.2 ± 4.6
Vertical Saccade Velocity Averaged	53.3 ± 14.9	48.9 ± 13.5
Vertical Saccade Amplitude	189.3 ± 20.8	189.8 ± 19.9
Horizontal Smooth Pursuit	93.2 ± 4.5	92.6 ± 4.7
Circular Smooth Pursuit Variance	13.8 ± 8.2	15.2 ± 8.3
Fixation Dispersion	6.8 ± 3.7	6.8 ± 3.8
Vertical Smooth Pursuit	95.8 ± 5.3	94.9 ± 5.7
Vertical Smooth Pursuit number of Saccades	5.3 ± 4.1	6.8 ± 4.4
Horizontal Smooth Pursuit number of Saccades	3.2 ± 3.0	3.9 ± 3.2
Horizontal Q Ratio	2.3 ± 0.3	2.3 ± 0.3
Vertical Q Ratio	2.4 ± 0.3	2.4 ± 0.3

Results are shown as *n* (%), mean ± SD, or mean ± SD [min–max].

**Table 2:** Technical definition and measurement of all variables.

Test Variable	Technical Definition and Measurement
Saccade Tests–Horizontal Amplitude	Average distance between consecutive turning points. Measured in millimeters (mm)
Saccade Tests–Horizontal Velocity	Refers to the average velocity made by the saccades across the test time. Measured in degrees per second (dps)
Saccade Tests–Vertical Amplitude	Average distance between consecutive turning points. Measured in millimeters (mm)
Saccade Tests–Vertical Velocity	Refers to the average velocity made by the saccades across the test time. Measured in degrees per second (dps)
Pursuit Tests–Horizontal saccade number	The total tally of saccades recorded during the pursuit. Measured as a count number
Pursuit Tests–Horizontal smooth pursuit	Refers to % of time spent in SP with acceptable distance and speed; 100% is perfect. Measured as a percentage (%)
Pursuit Tests–Vertical saccade number	The total tally of saccades recorded during the pursuit. Measured as a count number
Pursuit Tests–Vertical smooth pursuit	Refers to % of time spent in SP with acceptable distance and speed; 100% is perfect. Measured as a percentage (%)
Pursuit Tests–CircularVariability	Refers to the average variance from the ideal pathway. We look at variance in three segments of the pathway, middle, left/right or up/down
FixationFixation Dispersion	Refers to the average variance from the ideal pathway. We look at variance in three segments of the pathway, middle, left/right or up/down

**Table 3:** T-tests of eye movements by sex with Bonferroni correction.

	MM	SDM	FM	SDF	t	*p*	d [95% CI]
Horizontal Saccade Targeting	12.2	6.5	11.1	5.6	14.66	***	0.19 [0.17, 0.22]
Horizontal Saccade Amplitude	197.4	19.2	197.3	18.0	0.11	0.913	0.00 [−0.02, 0.03]
Horizontal Saccade Velocity	54.2	17.1	47.8	13.6	31.61	***	0.41 [0.39, 0.44]
Vertical Saccade Velocity	53.3	14.9	48.9	13.5	23.58	***	0.31 [0.28, 0.33]
Vertical Saccade Amplitude	189.3	20.8	189.8	19.9	−1.59	0.111	−0.02 [−0.05, 0.00]
Horizontal Smooth Pursuit	93.2	4.5	92.6	4.7	11.18	***	0.15 [0.12, 0.17]
Circular Smooth Pursuit Variance	13.8	8.2	15.2	8.3	−12.54	***	−0.16 [−0.19, −0.14]
Fixation Dispersion	6.8	3.7	6.8	3.8	−0.49	0.623	−0.01 [−0.03, 0.02]
Vertical Smooth Pursuit	95.8	5.3	94.9	5.7	12.70	***	0.17 [0.14, 0.19]
Vertical Smooth Pursuit number of Saccades	5.3	4.1	6.8	4.4	−27.80	***	−0.36 [−0.39, −0.34]
Horizontal Smooth Pursuit number of Saccades	3.2	3.0	3.9	3.2	−17.46	***	−0.23 [−0.25, −0.20]
Horizontal Q Ratio	2.3	0.3	2.3	0.3	5.21	***	0.07 [0.04, 0.09]
Vertical Q Ratio	2.4	0.3	2.4	0.3	−1.11	0.266	−0.01 [−0.04, 0.01]

MM = Male Median, FM = Female Median, SDM = Standard Deviation Male, SDF = Standard Deviation Female, *** *p* < 0.001, d = Cohen’s d.

**Table 4:** Multiple regression of eye movements by sex.

	Male	Female
Intercept	20.86 *** [9.27, 32.45]	7.18 [−5.24, 19.60]
Horizontal Saccade Targeting	0.23 *** [0.18, 0.28]	0.32 *** [0.25, 0.38]
Horizontal Saccade Amplitude	−0.03 ** [−0.05,−0.01]	0.04 *** [0.02, 0.07]
Horizontal Saccade Velocity	−0.14 *** [−0.16,−0.12]	−0.22 *** [−0.25, −0.18]
Vertical Saccade Targeting	0.10 ** [0.04, 0.17]	0.06 [−0.02, 0.14]
Vertical Saccade Velocity	−0.05 ** [−0.07, −0.02]	0.00 [−0.03, 0.04]
Vertical Saccade Amplitude	0.15 *** [0.13, 0.16]	0.11 *** [0.09, 0.13]
Horizontal Smooth Pursuit	0.00 [−0.06, 0.07]	0.05 [−0.02, 0.12]
Circular Smooth Pursuit Variance	−0.00 [−0.04, 0.03]	0.11 *** [0.07, 0.15]
Fixation Dispersion	−0.08 [−0.16, −0.00]	−0.34 *** [−0.42, −0.25]
Vertical Smooth Pursuit	0.05 [−0.02, 0.12]	0.01 [−0.06, 0.09]
Vertical Smooth Pursuit number of Saccades	−0.57 *** [−0.67, −0.48]	−0.21 *** [−0.31, −0.11]
Horizontal Smooth Pursuit number of Saccades	0.11 [−0.02, 0.23]	0.21 *** [0.08, 0.34]
Horizontal Q Ratio	−7.37 *** [−8.33, −6.42]	−4.85 *** [−5.93, −3.77]
Vertical Q Ratio	2.88 *** [1.95, 3.80]	2.00 *** [0.97, 3.03]
Number of Observations	11,871	11,686
R-squared	0.097	0.057
Adjusted R-squared	0.096	0.056

Results show coefficient with 95% CI in brackets, *** *p* < 0.001, ** *p* < 0.01.

## Data Availability

The data are not publicly available due to the expense and volume of cloud-based processes.
